# A preview of selected articles

**DOI:** 10.1002/sctm.20-0395

**Published:** 2020-09-26

**Authors:** Stuart P. Atkinson

**Affiliations:** ^1^ Centro de Investigación Príncipe Felipe Valencia Spain

## Abstract

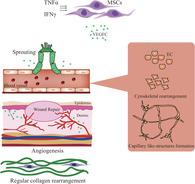

Members of the vascular endothelial growth factor (VEGF) family, a subfamily of the platelet‐derived growth factor family of cystine‐knot growth factors, act as signaling proteins that control both the de novo formation of the vasculature during development (vasculogenesis) and the growth of blood vessels from pre‐existing vasculature (angiogenesis). Successful wound healing or recovery from tissue ischemia requires the formation of new blood vessels to restore blood flow and supply the oxygen and nutrients required to support the growth and function of reparative cells.^1^ VEGF family members represent crucial regulators of this process by promoting the proliferation, migration, differentiation, and survival of endothelial cells from arteries, veins, and lymphatics. The family members, VEGF‐A, B, C, D, and placental growth factor, function by binding and activating specific tyrosine kinase receptors (VEGFR‐1, ‐2, and ‐3) to control the above‐mentioned processes. Interestingly, multiple studies have established that transplanted mesenchymal stem cells (MSCs) produce and secrete VEGF‐A to promote wound healing and recovery from hind limb ischemia by inducing angiogenesis in vivo^2^, ^3^; but do the other family members also play a role? In our first Featured Article published this month in *STEM CELLS Translational Medicine*, Zhu et al report that MSCs pretreated with inflammatory cytokines display enhanced angiogenic and wound‐healing capacities thanks to the expression of a specific VEGF family member protein.^4^ In a Related Article published recently in *STEM CELLS*, Yang et al described how the product of a short open reading frame within the 5′‐terminal noncoding area of Hdac7 mRNA in VEGF‐treated vascular progenitor cells induced vascular repair and angiogenesis in ischemic tissue.^5^

Myocardial infarction occurs when the blood flow, and hence oxygen supply, to part of the heart decreases or stops, leading to the permanent loss of cardiomyocytes, the formation of scar tissue, and the seemingly irreversible loss of cardiac function that can induce heart failure. Stem cell therapy aims to support cardiac homeostasis and the regeneration of lost cells/tissues in the adult heart through, in part, the provision of multiple growth factors and immune‐modulatory cytokines. Recent studies have provided yet more evidence that the transplantation of MSCs can support the repair of damaged cardiac tissue after myocardial infarction,^6^, ^7^, ^8^ with some preclinical investigations supporting the concept that MSCs derived from the heart itself, which are more oriented toward a cardiovascular phenotype, may favor better outcomes.^9^ Unfortunately, poor homing, low survival, and a lack of efficient engraftment in the hostile microenvironment of the injured heart represent significant obstacles to the overall efficacy of MSC therapy. However, the administration of MSCs overexpressing or pretreated with growth factors to improve therapeutic output and the implementation of enhanced MSC‐targeting strategies to improve homing may lead to significantly better outcomes. In our second Featured Article published this month in *STEM CELLS Translational Medicine*, Zhao et al report how the treatment of cardiac MSCs with growth differentiation factor 11 (GDF11) prompted improved cell survival, retention, and their overall therapeutic efficacy following their transplantation into the infarcted mouse heart.^10^ In a Related Article published recently in *STEM CELLS*, Chen et al described how cells engineered to target fibrin may represent an exciting means to improve MSC homing to the infarcted heart and induce greater therapeutic efficacy.^11^

## Featured Articles

### MSC‐Derived VEGF‐C Boosts Skin Wound Healing

Preconditioning MSCs can boost the secretion of paracrine‐acting factors such as fibroblast growth factor and VEGF‐A and, for example, improve skin wound healing.^12^, ^13^ As earlier studies from the laboratories of Guozhong Lv (The Third People's Hospital of Wuxi/Wuxi Medical College of Jiangnan University, Wuxi), Changshun Shao and Yufang Shi (Soochow University, Suzhou, China) previously reported that pro‐inflammatory stimuli induced the expression of a large number of secretory proteins from MSCs,^14^ they sought to evaluate this preconditioning step in the improvement of skin wound healing by MSCs in a murine model of cutaneous excision. Reporting in *STEM CELLS Translational Medicine*,^4^ Zhu et al established that the supernatant derived from MSCs pretreated with pro‐inflammatory stimuli (interferon‐gamma and tumor necrosis factor‐alpha) displayed an enhanced ability to promote angiogenesis, induce a more condensed and regular collagen rearrangement, and accelerate wound closure compared with a non‐preconditioned control MSC supernatant. Fascinatingly, the authors specifically highlighted the crucial role of elevated levels of VEGF‐C in the enhanced wound‐healing effects; indeed, the depletion of VEGF‐C in MSCs by RNA interference inhibited any beneficial effects on angiogenesis and wound healing while the addition of recombinant VEGF‐C protein recovered any lost function. Overall, this exciting new study provides support for the preconditioning of MSCs with pro‐inflammatory stimuli to enhance their paracrine capabilities and boost skin wound healing while also revealing a novel role for VEGF‐C in angiogenesis.

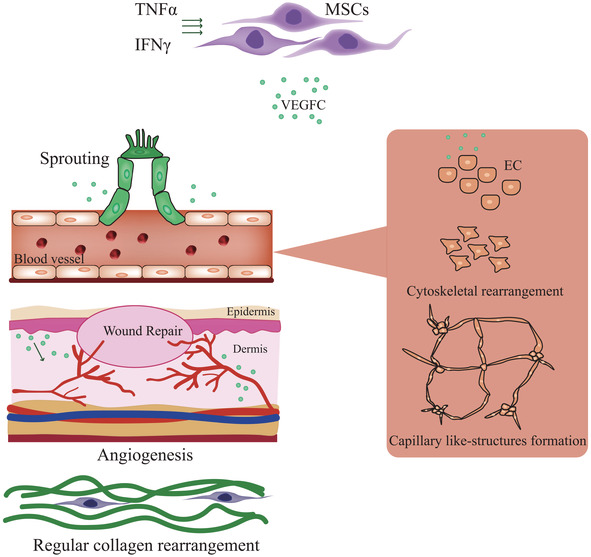

https://doi.org/10.1002/sctm.19-0241

### Boosting MSC Therapy for Myocardial Infarction With GDF11

Studies have established that GDF11, a member of the transforming growth factor‐β superfamily, stimulates the proliferation and angiogenesis of stem cells in ischemia/reperfusion models.^15^, ^16^ With this in mind, researchers led by Hong Yu (Zhejiang University, Hangzhou, Zhejiang Province, China) sought to explore GDF11 overexpression as a means to improve the viability and the therapeutic capacity of mouse cardiac MSCs with regard to the treatment of myocardial infarction. Reporting recently in *STEM CELLS Translational Medicine*,^10^ Zhao et al discovered that MSCs overexpressing GDF11 or pre‐treated with recombinant GDF11 displayed lower apoptosis, enhanced secretory abilities, and improved mitochondrial morphology and function when cultured under hypoxic conditions when compared to unmodified/untreated MSCs. At the molecular level, the authors established that GDF11 activated the activin receptor‐like kinase (ALK) 5 and SMAD2/3 signaling pathways, which then upregulated the expression of an ATP‐dependent metalloprotease (YME1L1) to balance the ratio of the long and short forms of processing of optic atrophy 1 (OPA1), a protein that controls fusion and fission in the inner mitochondrial membrane.^17^ Moving in vivo, the study reported improved cell survival and retention times following the transplantation of GDF11‐overexpressing MSCs into the infarcted heart, and encouragingly, these improvements prompted higher levels of angiogenesis, a reduction in scar formation, and an overall improvement in cardiac function when compared with control MSCs. The authors anticipate that their findings could pave the way for the development of enhanced stem cell therapies for cardiovascular diseases, including myocardial infarction, by targeting mitochondrial maintenance.

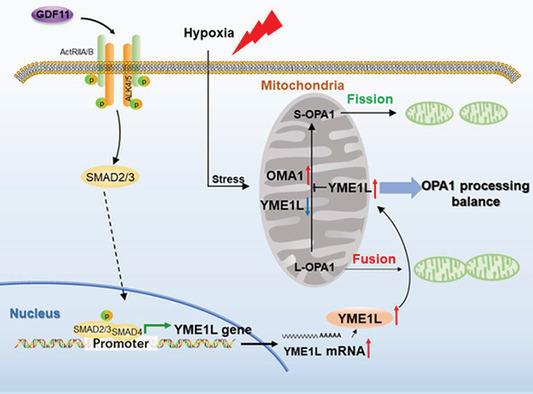

https://doi.org/10.1002/sctm.20-0005

## Related Articles

### How a VEGF‐Induced Peptide Induces Vascular Progenitor Cell‐Mediated Regeneration

Recent research has established that the expression of histone deacetylase 7 (HDAC7) in the vascular endothelium helps to maintain vascular integrity during early embryogenesis^18^, ^19^; therefore, a deeper understanding of HDAC7 function may permit the development of novel approaches to boost vascular repair and angiogenesis in ischemic tissue. Fascinatingly, a recent *STEM CELLS* study from Qian Wang (Southern Medical University, Guangzhou, Guangdong, China) and Lingfang Zeng (King's College London, London, UK) reported that VEGF‐A‐stimulated vascular progenitor cells produced a 7‐amino acid peptide (7A) from a short open reading frame within the 5′‐terminal noncoding area of a mouse Hdac7 transcript variant both in vitro and in vivo.^5^ Yang et al discovered that the 7A peptide functioned as a signal transducer by transferring phosphate groups from the activated mitogen‐activated protein kinase MEKK1 to the 14‐3‐3 gamma protein in response to VEGF stimulation. This step disrupts the cadherin‐plakoglobin‐catenin‐14‐3‐3γ complex and promotes the nuclear translocation of 14‐3‐3γ. Subsequent functional analysis established a role for the 7A peptide in the activation and migration of vascular progenitor cells, the re‐endothelialization of the injured femoral artery, and increased levels of angiogenesis in a hind limb ischemia model. Overall, this fascinating study provides evidence that small peptides derived from short open reading frames could play crucial roles in multiple physiological and pathological processes by acting as signal transducers.

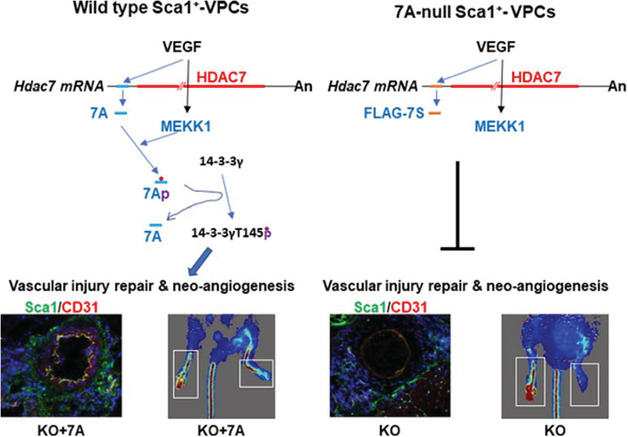

https://doi.org/10.1002/stem.3122

### Fibrin‐Targeting of MSCs Boosts MSC Therapy in the Infarcted Heart

Previous studies from researchers at Fudan University (Shanghai, China) had described fluorescently‐labeled superparamagnetic iron oxide nanoparticles conjugated with a homing peptide (cysteine‐arginine‐glutamic acid‐lysine‐alanine or CREKA) as a novel thrombus‐targeting agent for the multimodal imaging of microthrombi (microscopic clumps of fibrin, platelets, and red blood cells)^20^ and reported how a CREKA‐modified thymosin‐β4 accumulated in infarcted regions of the heart.^21^ Both studies took advantage of interactions between CREKA and fibrin, and the team followed‐up this exciting research by evaluating CREKA‐modified MSCs generated using liposome membrane fusion technology as a means to enhance fibrin‐mediated homing, induce functional recovery, and improve structural preservation in a rat myocardial injury model. In their recent *STEM CELLS* article,^11^ Chen et al described how CREKA‐modification enhanced the binding of MSCs to fibrin clots under static and flow conditions in vitro and, more excitingly, how CREKA‐modified MSCs accumulated in the injured rat myocardium to a greater extent than control MSCs and induced structural preservation and functional recovery due to the elevated levels of fibrin observed after myocardial injury. The authors note that a wide range of therapeutics may benefit from CREKA modification; indeed, the CREKA modification of drug combinations may promote synergism by ensuring the homing of modified factors to the required site within the same timeframe. Furthermore, as fibrin deposition occurs during the repair of almost all tissue injuries,^22^ CREKA may have therapeutic relevance further than myocardial infarction.

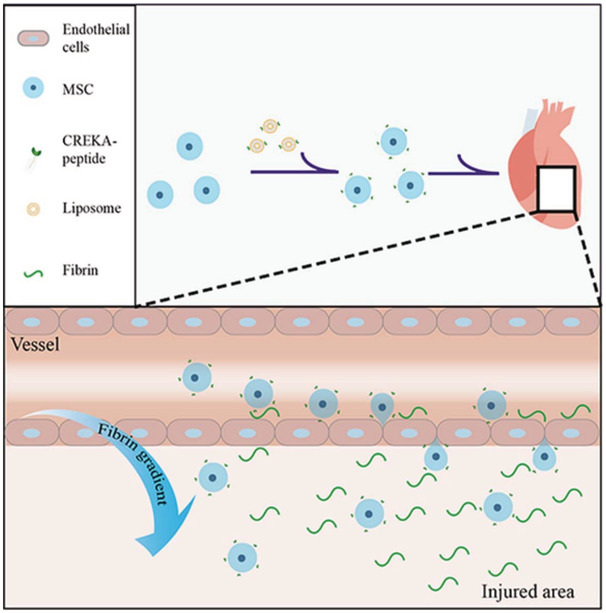

https://doi.org/10.1002/stem.2983
